# Mortality of Pregnancy Following Breast Cancer Diagnoses in Taiwanese Women

**DOI:** 10.1634/theoncologist.2019-0451

**Published:** 2019-11-25

**Authors:** Shu‐Chun Chuang, Ching‐Hung Lin, Yen‐Shen Lu, Chao Agnes Hsiung

**Affiliations:** ^1^ Institute of Population Health Sciences, National Health Research Institutes Zhunan Miaoli County Taiwan; ^2^ Department of Oncology, National Taiwan University Hospital Taipei Taiwan; ^3^ Department of Internal Medicine, National Taiwan University Hospital Taipei Taiwan

**Keywords:** Pregnancy, Breast cancer, Estrogen receptor, Survival

## Abstract

**Background:**

This work examined the association between pregnancy after breast cancer (BC) diagnosis and total mortality in Taiwanese patients with BC.

**Materials and Methods:**

The Taiwan Cancer Registry, National Health Insurance database, and Taiwan National Death Certificate database were reviewed. Patients who became pregnant after being diagnosed with BC were selected (*n* = 249). Four nonpregnant patients with BC were selected and matched to every pregnant patient with BC by age at diagnosis, year at diagnosis, and propensity score based on disease stage, tumor size, node involvement, and histological grade. The disease‐free time interval for the selected control needed to have been longer than the time interval between the cancer diagnosis and pregnancy for the index case. Follow‐up was calculated from the pregnancy date of the index case to the date of death or December 31, 2014, whichever came first. Cox proportional hazards models were used to estimate the hazard ratios (HRs) and 95% confidence intervals (CIs).

**Results:**

After adjusting for age, year at BC diagnosis, stage, positive nodes, and hormone therapy, patients with BC who became pregnant after their cancer diagnosis had lower total mortality than did the comparison group (HR = 0.44, 95% CI = 0.23–0.84), including that of estrogen receptor‐positive patients (HR = 0.23, 95% CI = 0.07–0.77). The inverse association was more pronounced for those who became pregnant more than 3 years after diagnosis (HR = 0.19, 95% CI = 0.05–0.78).

**Conclusion:**

Our nationwide retrospective analysis revealed that pregnancy after BC diagnosis was associated with lower mortality than that of nonpregnant patients with BC at a similar age, year at diagnosis, and clinical characteristics.

**Implications for Practice:**

This article provides high‐level evidence based on an Asian population for pregnancy counseling after a breast cancer diagnosis, including for patients with estrogen receptor‐positive cancers. The study also revealed the optimal time for patients who would like to become pregnant after breast cancer.

## Abstract 2

This article examines the association between pregnancy after breast cancer and total mortality in an Asian population, as well as explores the relationship between timing of the pregnancy and breast cancer survival.

## Introduction

Breast cancer incidence is increasing in East Asia, including Taiwan. Birth cohort exposures, including the rapid assimilation of the Western lifestyle by the more recent birth cohort, delayed childbearing, low parity, and reduced breastfeeding, are thought to be the major determinants of the increased incidence of breast cancer in younger generations in East Asian countries 1, 2, 3.

In Taiwan, the breast cancer incidence rate before age 50 was 8.4/100,000 in 1981–1985. The rate has increased to 31.2/100,000 in 2011–2015. In 2015, the median age at breast cancer diagnosis was 54 years, and approximately 36% of women were diagnosed before 50 years of age. Over the past decade, the 5‐year relative survival for breast cancer has increased from 83.9% in 2002–2006 to 88.1% in 2011–2015 4. However, the fertility rate decreased from 1.6 in 1999 to 1.1 in 2015, while the average age at first childbirth increased from 27 years in 1999 to 31 years in 2015 5. With the increasing incidence and improved prognosis for breast cancer, as well as delayed childbearing, physicians and care providers will likely see patients and families struggling between surviving cancer and building a family.

A prior study has explored the risk–benefit perceptions of pregnancy among breast cancer survivors in Taiwan 6, and “reaching the balance of life” was the core value when patients weighed the risks and gains of pregnancy. On one hand, the patients often became exhausted after chemotherapy and were uncertain about the safety of pregnancy for their health. They also worried about whether chemotherapy might harm the future baby. On the other hand, raising children would bring hope and happiness to their family. In Chinese culture, some women desire children because they expect their children to care for them when they become old, and this concept may be important for East Asian countries that share this culture of filial piety, such as Korea, Japan, Vietnam, and China.

In a subsequent study on fertility 7, 13 patients (70%) hoped to maintain reproductive function and 4 participants acquired relevant information about fertility preservation techniques and its pros and cons to the health of the mother and baby. None of the participants in this study took fertility‐preserving actions before treatment, which the authors suggested might be because of the possible invasive damage to the participants’ physical health and threat to their life if they delayed cancer treatment. Forgoing fertility preservation to have a better chance of survival was the major reason. In both these studies 6, 7, the authors indicated that a high level of evidence is necessary for more effective pregnancy counseling for women who wish to conceive after being diagnosed with breast cancer.

Currently, most epidemiological data confirm that pregnancy after breast cancer is safe for mothers 8, 9, 10, 11, even for those with estrogen receptor‐positive (ER+) cancers 12, 13, 14. In general, clinicians recommend that patients wait at least 2 years after their diagnosis before getting pregnant 10, 11. A recent study suggested that the timing can be shortened to 6 months after the diagnosis 15. Most of these studies were conducted in the U.S. or Europe; however, to our knowledge, no published studies have reported the safety of pregnancy after breast cancer diagnosis in Asian populations.

Therefore, we conducted this study to examine the association between pregnancy after breast cancer and total mortality in an Asian population. We also explored the relationship between the timing of the pregnancy and breast cancer survival.

## Materials and Methods

### Study Population

The study population was selected as described previously 16. Briefly, patients with breast cancer were selected from the Taiwan Cancer Registry (TCR). The inclusion criteria were (a) stage I–III first primary invasive breast cancer; (b) age at diagnosis between 20 and 50 years; and (c) diagnosed between 2002 and 2014. Tumor characteristics (stage, size, positive nodes, histological grade, estrogen receptor [ER] status, progesterone receptor [PR] status, and human epidermal growth receptor 2 [HER2] status), types and dates of the treatments (surgery, radiotherapy, chemotherapy, and hormone therapy), recurrence status, and date of recurrence were recorded (*n* = 30,479). The pregnancy records, delivery outcomes, and chemotherapy drugs were retrieved from the Taiwan National Health Insurance (NHI) database. A pregnancy event was defined as having prenatal examinations or a history of delivery or abortion. The prenatal examinations were covered by the NHI program, and 98% of mothers underwent at least four examinations 17. Pregnancy dates were calculated from the prenatal examination dates and the dates of delivery or abortion.

Patients with breast cancer who became pregnant after their cancer diagnosis (BCPPs) were selected (*n* = 249). Patients who were diagnosed during pregnancy or had a recent pregnancy record of <5 years before their cancer diagnosis were excluded (*n* = 2,430). A propensity score for pregnancy was created according to disease stage, tumor size, node involvement, and histological grade. Four comparison patients with no pregnancy records for 5 years prior to, during, and after cancer diagnosis were selected from the remaining patients and matched to each BCPP. The comparison patients were matched to the BCPPs on age at diagnosis (±2 years), year at diagnosis (±1 year), and propensity score for pregnancy. To control for the healthy mother effect, comparison patients must have had a longer disease‐free time interval than the time interval between cancer diagnosis and pregnancy of the index BCPPs. Comparison patients were assigned an index date, which was defined as the pregnancy date of the index BCPPs. Disease‐free status was checked using the recurrence status and date in the TCR and the following criteria from the NHI database: (a) those with ER‐negative (ER−) tumors and who began chemotherapy 1 year after diagnosis, or (b) those with ER+ tumors and who had changed chemotherapy drugs. These criteria were discussed with the collaborative doctors (C.‐H.L. and Y.‐S.L.) based on their experience in practice. Selected comparison patients with any of these conditions before the index date were removed from the comparison group. The final data included 249 pregnant and 914 nonpregnant patients.

The study was based on secondary data analysis. The clinical characteristics of the diagnosed cancer and treatment for the selected patients were retrieved from the TCR and NHI databases. The vital status, causes, and date of death were retrieved from the Taiwan National Death Certificate database. The ethical review board of the National Health Research Institutes, Taiwan, approved the study.

### Statistical Analysis

The length of follow‐up was calculated from the pregnancy date (or index date for the controls) to the date of death or December 31, 2014, whichever came first. Multivariate Cox proportional hazards models were used to estimate hazard ratios (HRs) and 95% confidence intervals (CIs) for the association between pregnancy and overall survival. The confounding factors under consideration were age at breast cancer diagnosis, year of breast cancer diagnosis, stage, tumor size, lymph node involvement, histological grade, ER status, PR status, surgery types, radiotherapy, chemotherapy, and hormone therapy. Because no HER2 status could be determined for 89% of the selected patients, HER2 status was not assessed as a potential confounder. However, when all variables were included in the model, only age at breast cancer diagnosis, year of breast cancer diagnosis, stage, lymph node involvement, and hormone therapy remained statistically significant at α = 0.1. Thus, only these five variables were used in the final model. Because age at diagnosis can directly affect overall survival, we adjusted for age using stratification.

Analyses were performed using SAS 9.4 (SAS Institute, Cary, NC). All tests were two‐sided, and statistical significance was considered at *p* < .05.

## Results

Table 1 shows the clinical characteristics of the BCPPs and comparison patients. After propensity score matching, the BCPP and comparison group had similar disease stages, tumor size, node involvement, and histological grades. For those with a known ER status, 65.4% in the BCPP group and 68.5% in the comparison group were ER+ (*p* = .50). The BCPP were more likely than were the comparison patients to have had conservative breast treatments and less likely to have had chemotherapy or hormone therapy. Overall, 12 BCPPs (4.8%) and 69 comparison group patients (7.5%) died during the observation period.

**Table 1:**
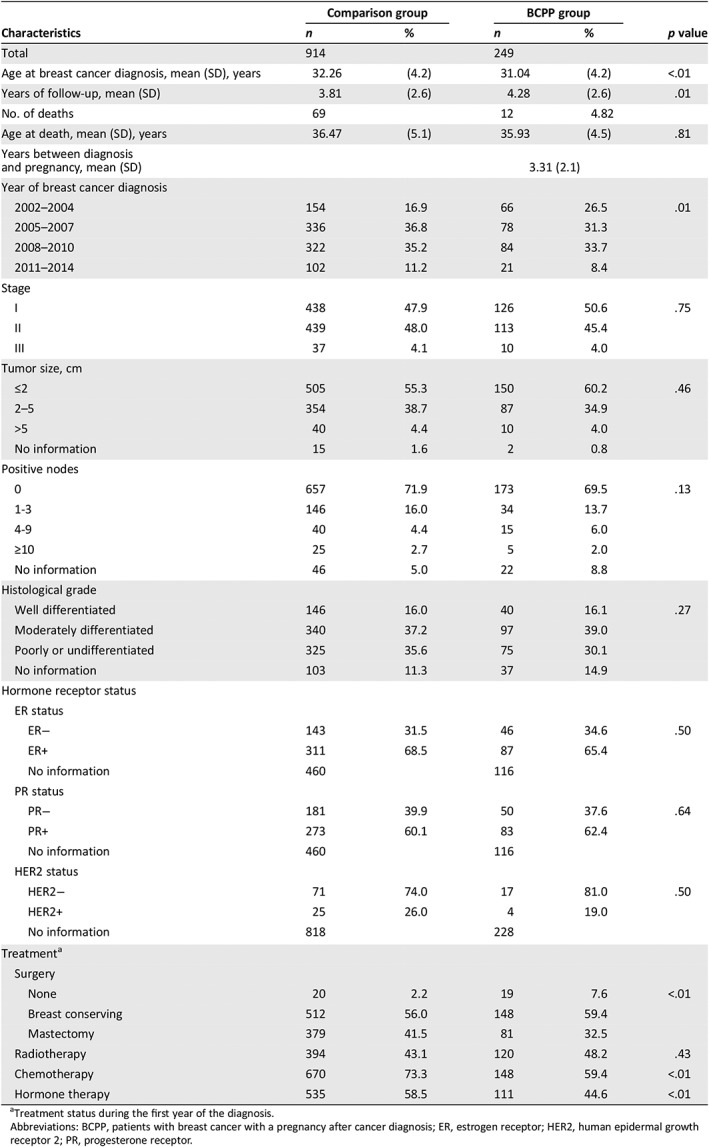
Characteristics of BCPPs and the comparison group

Characteristics	Comparison group		BCPP group		*p* value
	*n*	%	*n*	%	
Total	914		249		
Age at breast cancer diagnosis, mean (SD), years	32.26	(4.2)	31.04	(4.2)	<.01
Years of follow‐up, mean (SD)	3.81	(2.6)	4.28	(2.6)	.01
No. of deaths	69		12	4.82	
Age at death, mean (SD), years	36.47	(5.1)	35.93	(4.5)	.81
Years between diagnosis and pregnancy, mean (SD)			3.31 (2.1)		
Year of breast cancer diagnosis					
2002–2004	154	16.9	66	26.5	.01
2005–2007	336	36.8	78	31.3	
2008–2010	322	35.2	84	33.7	
2011–2014	102	11.2	21	8.4	
Stage					
I	438	47.9	126	50.6	.75
II	439	48.0	113	45.4	
III	37	4.1	10	4.0	
Tumor size, cm					
≤2	505	55.3	150	60.2	.46
2–5	354	38.7	87	34.9	
>5	40	4.4	10	4.0	
No information	15	1.6	2	0.8	
Positive nodes					
0	657	71.9	173	69.5	.13
1‐3	146	16.0	34	13.7	
4‐9	40	4.4	15	6.0	
≥10	25	2.7	5	2.0	
No information	46	5.0	22	8.8	
Histological grade					
Well differentiated	146	16.0	40	16.1	.27
Moderately differentiated	340	37.2	97	39.0	
Poorly or undifferentiated	325	35.6	75	30.1	
No information	103	11.3	37	14.9	
Hormone receptor status					
ER status					
ER−	143	31.5	46	34.6	.50
ER+	311	68.5	87	65.4	
No information	460		116		
PR status					
PR−	181	39.9	50	37.6	.64
PR+	273	60.1	83	62.4	
No information	460		116		
HER2 status					
HER2−	71	74.0	17	81.0	.50
HER2+	25	26.0	4	19.0	
No information	818		228		
Treatment^a^					
Surgery					
None	20	2.2	19	7.6	<.01
Breast conserving	512	56.0	148	59.4	
Mastectomy	379	41.5	81	32.5	
Radiotherapy	394	43.1	120	48.2	.43
Chemotherapy	670	73.3	148	59.4	<.01
Hormone therapy	535	58.5	111	44.6	<.01

a Treatment status during the first year of the diagnosis.

Abbreviations: BCPP, patients with breast cancer with a pregnancy after cancer diagnosis; ER, estrogen receptor; HER2, human epidermal growth receptor 2; PR, progesterone receptor.

**Figure 1:**
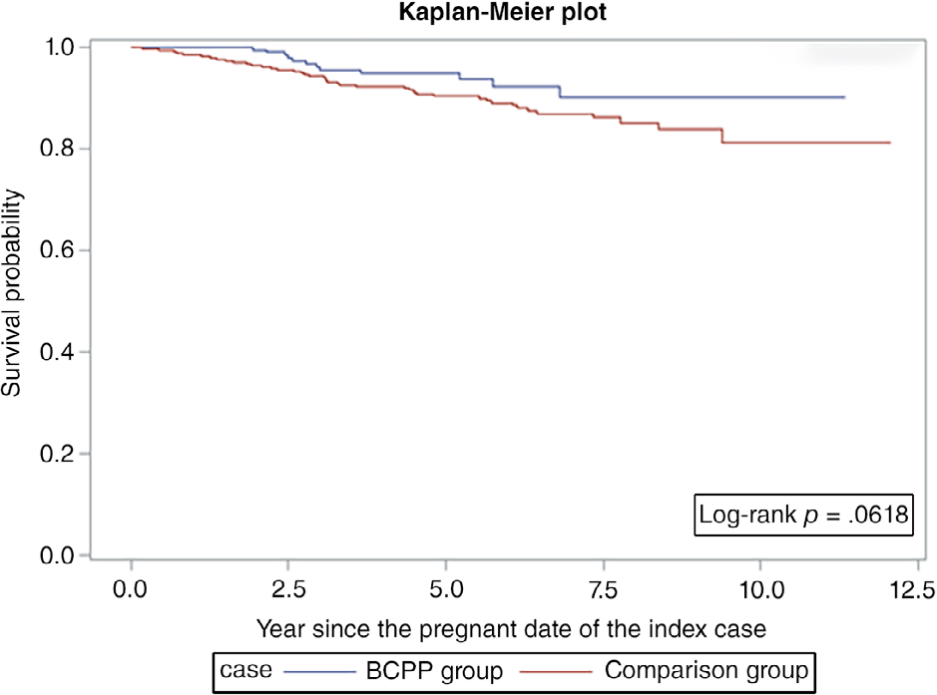
Survival curves for BCPPs and the comparison group.Abbreviation: BCPP, patients with breast cancer who became pregnant after their cancer diagnosis.

**Figure 2:**
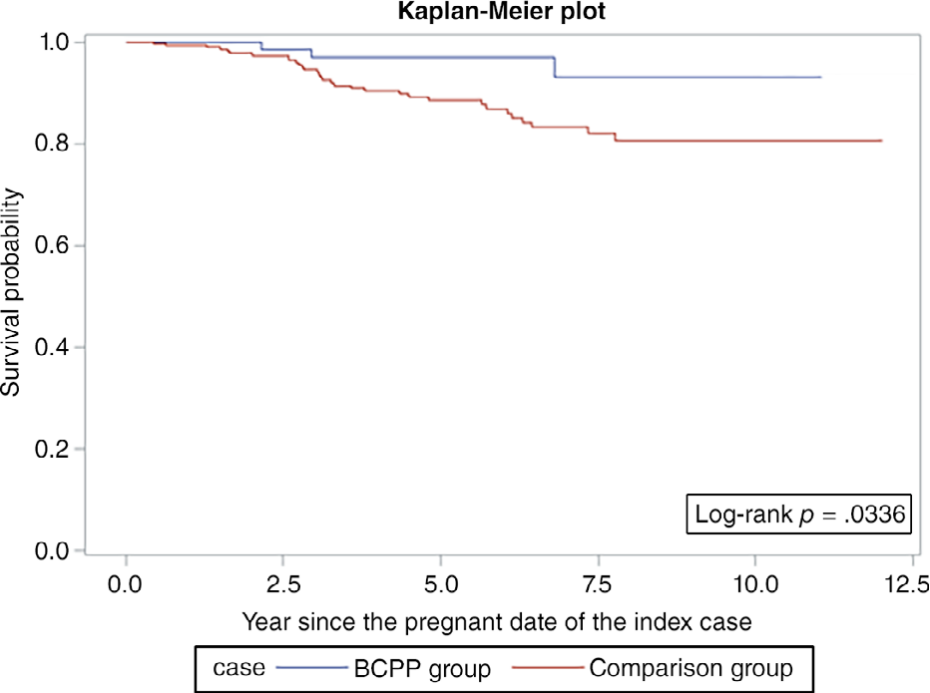
Survival curves for patients with estrogen receptor‐positive breast cancer who became pregnant after cancer diagnosis and the comparison group.Abbreviation: BCPP, patients with breast cancer who became pregnant after their cancer diagnosis.

**Figure 3:**
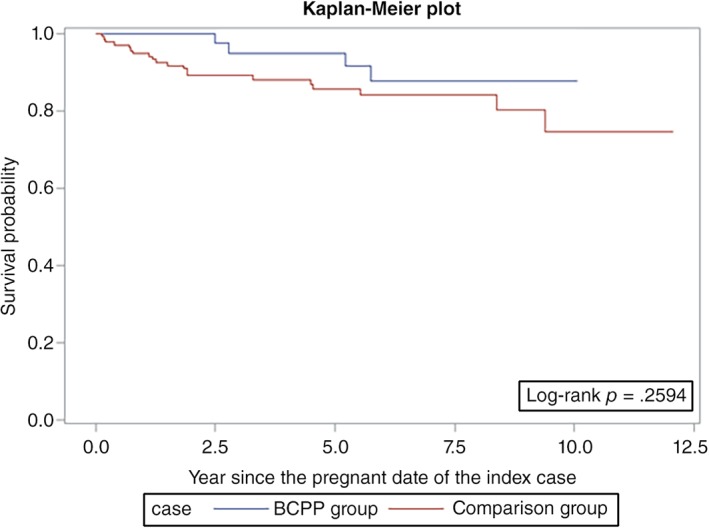
Survival curves for patients with estrogen receptor‐negative breast cancer who became pregnant after cancer diagnosis and the comparison group.Abbreviation: BCPP, patients with breast cancer who became pregnant after their cancer diagnosis.

Overall survival was similar among the BCPPs and the comparison group patients (log‐rank *p* = .06; Fig. 1). However, the differences were more evident in ER+ patients (log‐rank *p* = .03; Fig. 2) but not statistically significant for ER− patients (log‐rank *p* = .26; Fig. 3). After adjusting for age, year at breast cancer diagnosis, stage, positive nodes, and hormone therapy, BCPP had lower total mortality than did the comparison group (HR = 0.44, 95% CI = 0.23–0.84; Table 2). The inverse association was particularly evident for those who had waited to become pregnant for 3 years or more after the breast cancer diagnosis (HR = 0.19, 95% CI = 0.05–0.78). In addition, those who completed their pregnancy had lower mortality (HR = 0.40, 95% CI = 0.20–0.82), whereas patients who had spontaneous or induced abortions had similar mortality rates to those of the comparison group (HR = 0.73, 95% CI = 0.17–3.03).

**Table 2:**
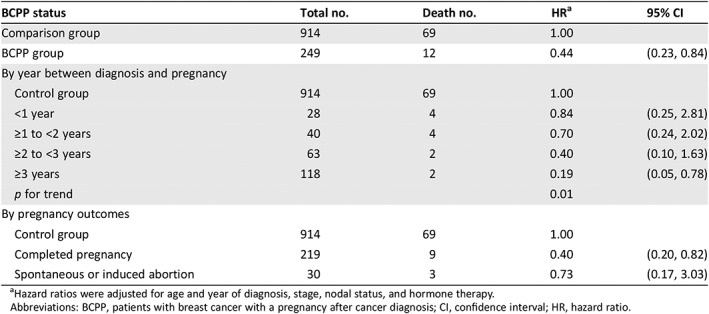
Association between pregnancy after breast cancer diagnosis and total mortality

BCPP status	Total no.	Death no.	HR^a^	95% CI
Comparison group	914	69	1.00	
BCPP group	249	12	0.44	(0.23, 0.84)
By year between diagnosis and pregnancy				
Control group	914	69	1.00	
<1 year	28	4	0.84	(0.25, 2.81)
≥1 to <2 years	40	4	0.70	(0.24, 2.02)
≥2 to <3 years	63	2	0.40	(0.10, 1.63)
≥3 years	118	2	0.19	(0.05, 0.78)
*p* for trend			0.01	
By pregnancy outcomes				
Control group	914	69	1.00	
Completed pregnancy	219	9	0.40	(0.20, 0.82)
Spontaneous or induced abortion	30	3	0.73	(0.17, 3.03)

a Hazard ratios were adjusted for age and year of diagnosis, stage, nodal status, and hormone therapy.

Abbreviations: BCPP, patients with breast cancer with a pregnancy after cancer diagnosis; CI, confidence interval; HR, hazard ratio.

Pregnancy was generally associated with lower total mortality in all subgroup analyses (Table 3), but the evidence was more pronounced in patients who were younger than 30 years (HR = 0.29, 95% CI = 0.10–0.88), were at stage II or III (HR = 0.37, 95% CI = 0.16–0.82), had ER+ disease (HR = 0.23, 95% CI = 0.07–0.77), had received chemotherapy (HR = 0.40, 95% CI = 0.18–0.90), or did not receive hormone therapy (HR = 0.40, 95% CI = 0.17–0.91).

**Table 3:**
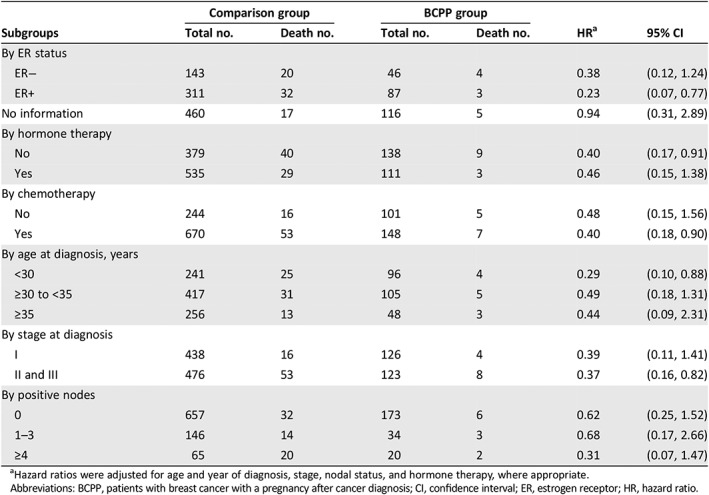
Subgroup analysis of the association between pregnancy after breast cancer diagnosis and total mortality

	Comparison group		BCPP group			
Subgroups	Total no.	Death no.	Total no.	Death no.	HR^a^	95% CI
By ER status						
ER−	143	20	46	4	0.38	(0.12, 1.24)
ER+	311	32	87	3	0.23	(0.07, 0.77)
No information	460	17	116	5	0.94	(0.31, 2.89)
By hormone therapy						
No	379	40	138	9	0.40	(0.17, 0.91)
Yes	535	29	111	3	0.46	(0.15, 1.38)
By chemotherapy						
No	244	16	101	5	0.48	(0.15, 1.56)
Yes	670	53	148	7	0.40	(0.18, 0.90)
By age at diagnosis, years						
<30	241	25	96	4	0.29	(0.10, 0.88)
≥30 to <35	417	31	105	5	0.49	(0.18, 1.31)
≥35	256	13	48	3	0.44	(0.09, 2.31)
By stage at diagnosis						
I	438	16	126	4	0.39	(0.11, 1.41)
II and III	476	53	123	8	0.37	(0.16, 0.82)
By positive nodes						
0	657	32	173	6	0.62	(0.25, 1.52)
1–3	146	14	34	3	0.68	(0.17, 2.66)
≥4	65	20	20	2	0.31	(0.07, 1.47)

a Hazard ratios were adjusted for age and year of diagnosis, stage, nodal status, and hormone therapy, where appropriate.

Abbreviations: BCPP, patients with breast cancer with a pregnancy after cancer diagnosis; CI, confidence interval; ER, estrogen receptor; HR, hazard ratio.

## Discussion

Among the 30,479 female patients with breast cancer in Taiwan, 249 became pregnant after their cancer diagnosis, representing a < 1% prevalence in patients aged 20–50 years diagnosed between 2002 and 2014. The BCPP had lower mortality than did their matched controls (HR = 0.44, 95% CI = 0.23–0.84), and the ER+ patients (HR = 0.23, 95% CI = 0.07–0.77). The inverse association was more pronounced for those who waited more than 3 years after their breast cancer diagnosis (HR = 0.19, 95% CI = 0.05–0.78).

The safety of pregnancy for patients with breast cancer has been confirmed in several epidemiological observational studies and meta‐analyses 8, 9, 10, 11, 18. A meta‐analysis of 14 studies demonstrated a 41% reduced risk of death for pregnant women with a history of breast cancer 11. Another meta‐analysis that pooled results from nine studies that used matching to control for “healthy mother effects” suggested a hazard ratio for death of 0.51 (95% CI = 0.42–0.62) 18. Nevertheless, another small‐scale observational study suggested that mental health, rather than physical health, may play a role in the healthy mother effects 19. However, safety remains a concern for ER+ patients. A U.S. study on patients with ER+ breast cancer found no difference in the 5‐year disease‐free survival rate between pregnant and nonpregnant cohorts (*p* = .34) 12. Another international multicenter study 13, 14 further demonstrated no disease‐free survival differences between pregnant and nonpregnant cohorts in both ER+ (HR = 0.94, 95% CI = 0.70–1.26) and ER− patients (HR = 0.75, 95% CI = 0.53–1.06). The latter study 14 also suggested statistically significant better overall survival in the pregnant cohort for ER− patients (HR = 0.57, 95% CI = 0.36–0.90).

In our study, we observed a better overall survival in the pregnant cohort for ER+ patients and no difference for ER− patients (Figs. 2, 3; Table 3). Although we attempted to control for the healthy mother effect by matching the propensity score and time to pregnancy in the control selection, the healthy mother effect may remain a concern if a prepregnancy evaluation was performed 20. The healthy mother effect may be stronger in ER+ patients, because the detrimental effects of hormone simulation during pregnancy are of particular concern for ER+ patients. Nevertheless, studies on breast cancer during pregnancy have also suggested that maternal immunity is stimulated against cancer cells during pregnancy, which is known as the “fetal antigen hypothesis” 21. Furthermore, the high levels of estrogen, progesterone, and human gonadotropin during pregnancy may induce apoptosis in endocrine‐responsive breast cancer cells 22, 23, 24. These hypotheses suggest that pregnancy might not be detrimental for patients with a history of breast cancer.

For a patient with breast cancer who wishes to conceive, the current recommendation is to wait for at least 2 years from the time of breast cancer diagnosis 10, 11. This recommendation was based on positive results from observational studies of patients who became pregnant after breast cancer, the high incidence of tumor recurrence during the first 2 years 25, and the time window that allows patients to recover from chemotherapy‐induced ovarian toxicity 26. Our study suggested that the risk of death decreased as patients waited longer between diagnosis and pregnancy (*p* = .01), particularly for those who waited more than 3 years (Table 2). The abortion rate in our cohort was approximately 12% (30/249), which was lower than that in studies from Western countries 12, 13, 27, 28. This may be because of the cultural stigma concerning infertility and the traditional value of family in Taiwan. Patients who strongly wanted a child would attempt to conceive after cancer treatment regardless, whereas those who were hesitant or believed pregnancy to be harmful for survival may have decided against becoming pregnant 7. Nevertheless, no evidence suggested that spontaneous or induced abortion was associated with increased or decreased mortality in our study.

Our study had some limitations. First, although we ensured the disease‐free status for the selected comparison group, we could not completely rule out the possibility of the healthy mother effect. Second, the recurrence status might not have been completed in the cancer registry; however, we tried to amend this limitation using additional data from the drug database of the NHI. Third, half of our patients lacked an ER status in their record. However, the data still revealed an inverse association between a subsequent pregnancy and total mortality in ER+ patients. Finally, although we included a nationwide database, events remained limited over the 13‐year observation period. However, this may reassure patients that pregnancy is safe after breast cancer.

Currently, no guideline exists against pregnancy after breast cancer in Western countries. The American Society of Clinical Oncology indicates that pregnancy after cancer treatment is safe for both mothers and babies, and pregnancy does not increase the risk of recurrence. However, the wait time depends on the cancer type and stage, the treatment type, the need for ongoing treatment, age, and personal preferences 29. The National Comprehensive Cancer Network clinical practice guidelines in oncology suggest that all premenopausal patients should be counseled regarding their desire for future pregnancy. Patients who desire future pregnancy should be referred to fertility specialists before cancer treatment 30. The European Society for Medical Oncology does not discourage pregnancy after breast cancer irrespective of ER status. Inducing abortion does not affect breast cancer prognosis; thus, it is discouraged for purposes of attempting to change the cancer prognosis 31.

## Conclusion

In general, our conclusions are consistent with the aforementioned guidelines. We observed that subsequent pregnancy and total mortality were inversely associated in patients with breast cancer, even ER+ patients. Patients who completed pregnancy also had lower total mortality. The evidence was more pronounced for those who waited more than 3 years after their cancer diagnosis to become pregnant. Our nationwide retrospective analysis indicated that pregnancy after a breast cancer diagnosis was associated with lower mortality compared with that in a group of matched patients with similar ages, years at diagnosis, and clinical characteristics in Taiwan.

## Author Contributions

**Conception/design:** Ching‐Hung Lin, Yen‐Shen Lu

**Provision of study material or patients:** Shu‐Chun Chuang, Chao Agnes Hsiung

**Collection and/or assembly of data:** Shu‐Chun Chuang, Chao Agnes Hsiung

**Data analysis and interpretation:** Shu‐Chun Chuang, Ching‐Hung Lin, Yen‐Shen Lu, Chao Agnes Hsiung

**Manuscript writing:** Shu‐Chun Chuang, Ching‐Hung Lin, Yen‐Shen Lu, Chao Agnes Hsiung

**Final approval of manuscript:** Shu‐Chun Chuang, Ching‐Hung Lin, Yen‐Shen Lu, Chao Agnes Hsiung

## Disclosures

The authors indicated no financial relationships.
